# Complex Pediatric Elbow Injury: An Uncommon Case

**DOI:** 10.1186/1471-2474-6-13

**Published:** 2005-03-09

**Authors:** H Sharma, R Ayer, GR Taylor

**Affiliations:** 1 Department of Trauma and Orthopaedics, Victoria Infirmary, Glasgow, G42 9TY, UK; 2 Department of Trauma and Orthopaedics, Southampton General Hospital, Southampton, SO16 6YD, UK

**Keywords:** Complex pediatric elbow trauma, Lateral condyle fracture, Elbow dislocation.

## Abstract

**Background:**

There is paucity of literature describing complex elbow trauma in the pediatric population. We described a case of an uncommon pediatric elbow injury comprised of lateral condyle fracture associated with posterolateral dislocation of elbow.

**Case presentation:**

A 12-year-old boy sustained a direct elbow trauma and presented with Milch type II lateral condyle fracture associated with posterolateral dislocation of elbow. Elbow dislocation was managed by closed reduction. The elbow stability was assessed under general anaesthesia, followed by open K-wiring for the lateral condylar fracture fixation. The patient had an uneventful recovery with an excellent outcome at 39 months follow-up.

**Conclusion:**

Complex pediatric elbow injuries are quite unusual to encounter, the management of such fractures can be technically demanding. Concomitant elbow dislocation should be managed by closed reduction followed by open reduction and internal fixation (K-wires or cannulated screws) of the lateral condyle fracture.

## Background

Traumatic elbow dislocation is a rare injury in children constituting 3–6% of all elbow injuries [1]. It more frequently occurs with medial epicondyle fractures, although, it can infrequently be associated with lateral humeral condyle fracture [1-3]. The complex elbow anatomy with multiple growth centres appearing at different time period in the skeletally immature age can pose a diagnostic dilemma. The management of such unusual injuries can be technically demanding.

There is limited evidence available in the literature describing complex elbow fracture dislocation in the pediatric population [3-7]. This report presents a rare condition of a complex elbow injury consisting of lateral condyle fracture in association with posterolateral elbow dislocation. The patient had an excellent outcome after a follow-up of 39 months.

## Case presentation

**Figure 1:**
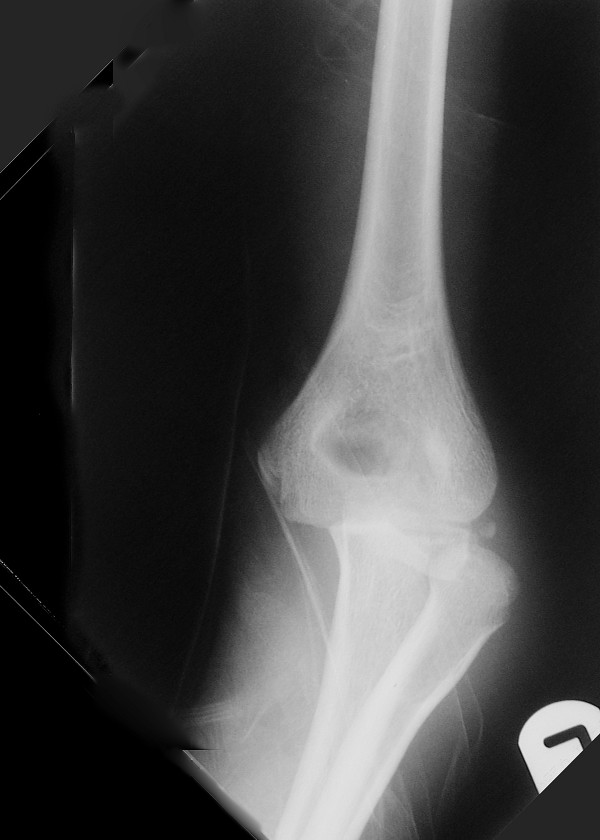
Preoperative anteroposterior radiograph of the elbow revealing fracture lateral condyle in association with posterolateral dislocation of elbow.

**Figure 2:**
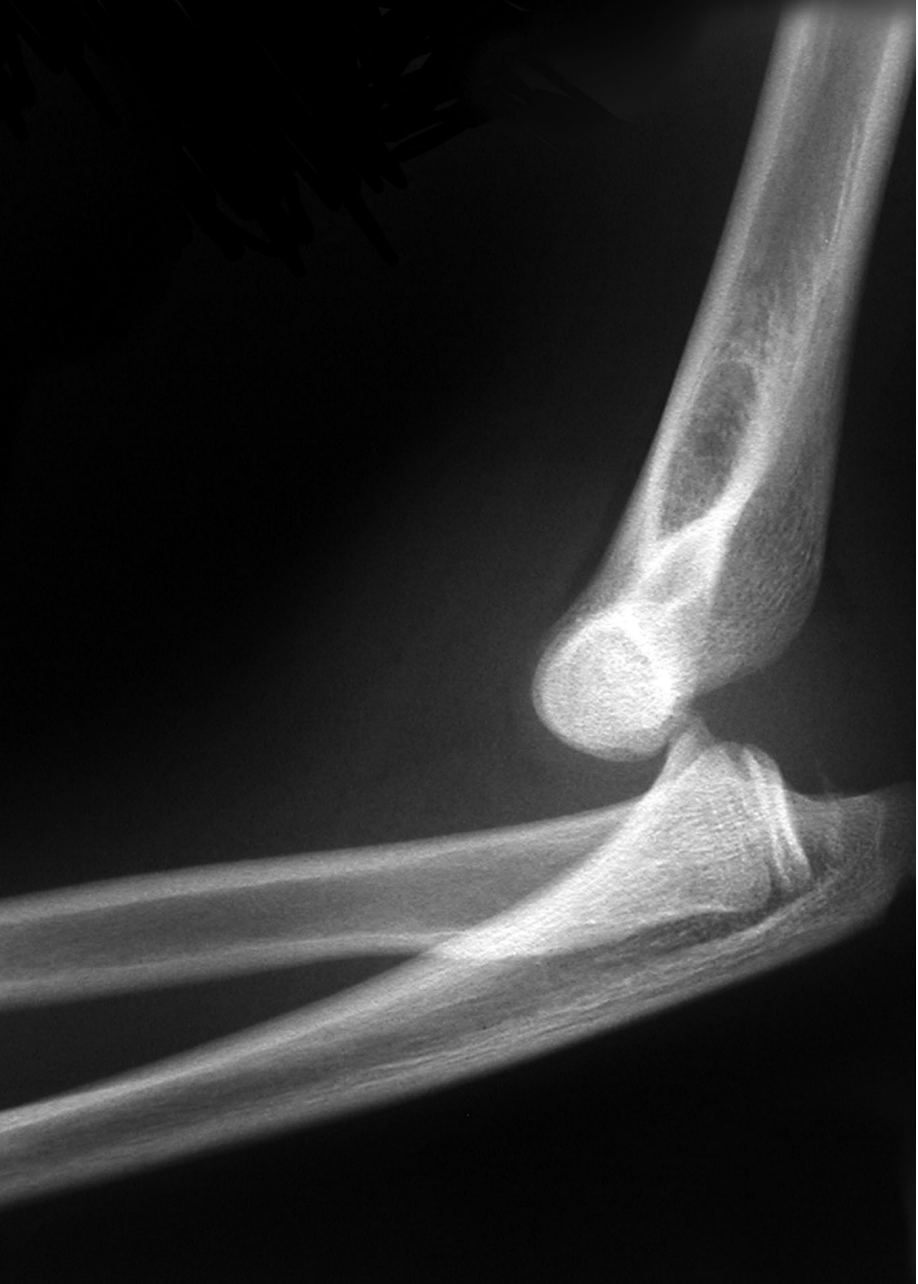
Preoperative lateral radiograph of the elbow revealing elbow dislocation.

A 12-year-old boy presented with a grossly swollen and deformed left elbow after sustaining a fall off skateboard and directly landed on the elbow. There was no neurovascular deficit in the extremity. The anteroposterior and lateral radiographs showed posterolateral dislocation of the left elbow in association with Milch type II lateral condyle fracture (Figure 1 and Figure 2). Concomitant elbow dislocation was managed by closed reduction. The stability of elbow was assessed under general anaesthesia.

**Figure 3:**
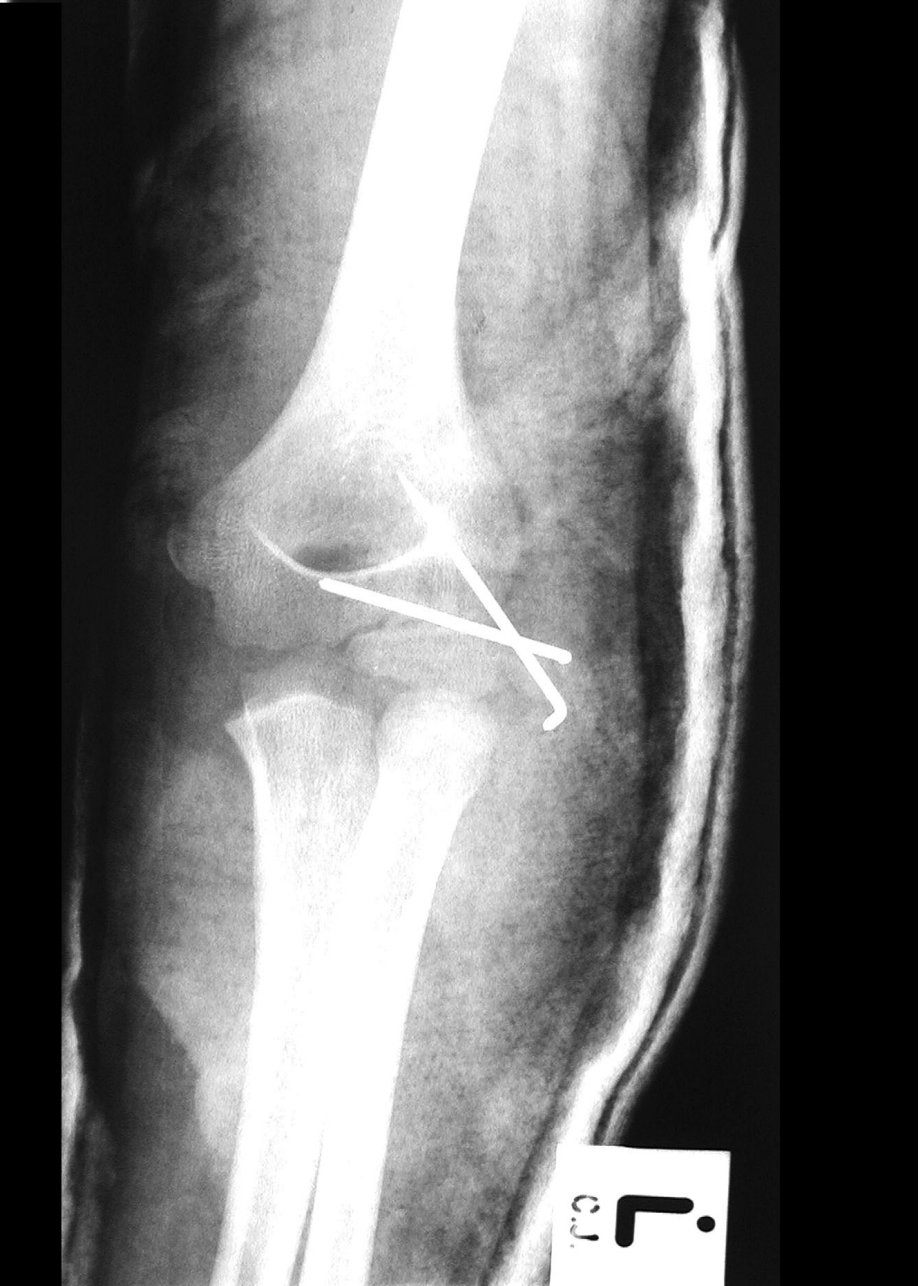
Postoperative anteroposterior radiograph of the elbow revealing internal fixation of the lateral condyle fracture with the help of 2 K-wires. The elbow dislocation was reduced first by closed technique.

**Figure 4:**
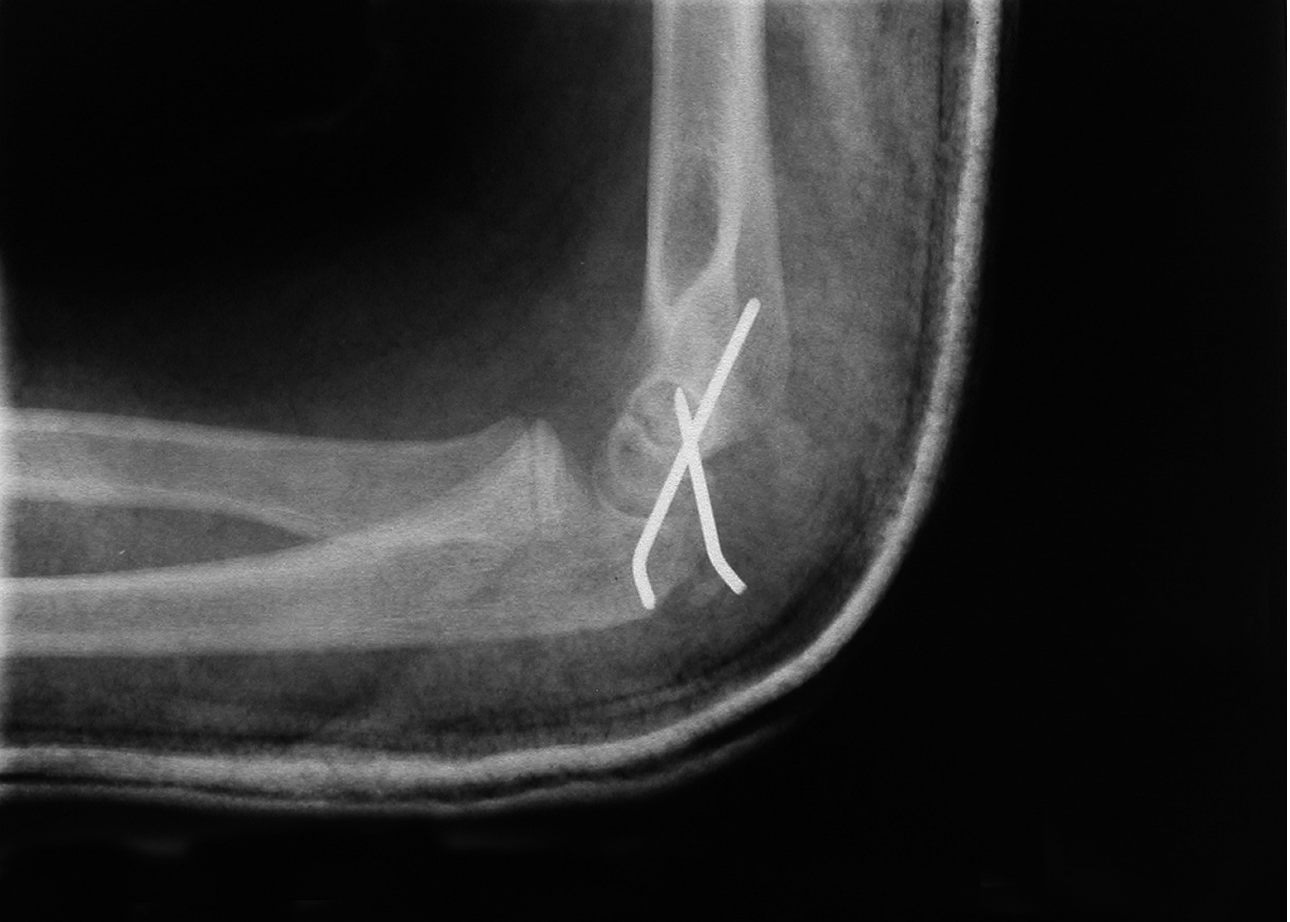
Postoperative lateral radiograph of the elbow revealing internal fixation of the lateral condyle fracture with the help of 2 K-wires.

Open K-wiring of the lateral condylar fracture was carried out on the same day of injury (Figure 3 and Figure 4). A standard lateral approach to the distal humerus and elbow joint was used. Under direct visualization, the fracture was anatomically reduced and held in place. Two smooth 0.0625-inch Kirschner wires (K-wires) were inserted from lateral to medial and distal to proximal directions. The positions of the K-wires were verified by fluoroscopic examination in the anteroposterior and lateral planes. The K-wires were then cut and left exposed outside the skin.

The operated elbow was immobilized in an above elbow backslab postoperatively. A close clinico-radiological follow-up at one and two weeks postoperatively was instituted and confirmed no loss of reduction. K-wires were removed under general anaesthesia four and half weeks postoperatively. At 39 months follow up, the elbow had normal appearance and functions with no alteration in the carrying angle and no symptoms.

## Discussion

Complex elbow injury pattern consisting of lateral condyle fracture in association with elbow dislocation has not been well-described in children. We found only a few cases described previously in the English literature. Lateral condyle fracture has been previously described in association with posterolateral elbow dislocation [3,5] and posteromedial elbow dislocation [4,8]. Posteromedial dislocation of the elbow with associated intraarticular entrapment of the lateral epicondyle has also been documented [2].

Isolated traumatic dislocation of the elbow is a rare injury in children constituting 3–6% of all elbow injuries. The peak incidence occurs in the thirteen to fourteen years of age. Dislocation of the elbow can be classified by the direction of the dislocation of the radius and ulna. Elbow dislocations are rarely associated with lateral condyle fractures, and more frequently occur with medial epicondyle fractures [1-3,9].

Lateral condyle fractures represent approximately 15 percent of all elbow fractures in children. It occurs more commonly between five and ten years of age. The lateral condyle is fractured by a varus stress applied to the extended elbow with the forearm supinated, as in falling on an outstretched hand. In addition, it can secondarily be fractured by the pull of the lateral collateral ligament and the extensor muscles [8,10].

There are two classifications which are currently in use to describe lateral condylar fractures. The Milch classification is based on the anatomical position of the fracture line. In Type I fracture, the fracture line courses lateral to the trochlea and passes through the capitello-trochlear groove. In the Type II injury, the fracture line extends into the apex of the trochlea. Milch described the more common Type II injury as a fracture-dislocation and the Type I injury as a simple fracture [11]. Lateral condyle fractures are also described in relation to the degree of displacement and rotation of the fracture fragment [12]. Stage I displaced fractures have less than two millimeters of displacement with intact articular surface. In Stage II displaced fractures, there is two to four millimeters of displacement with moderate displacement of the articular surface. Stage III displaced fractures demonstrate significant displacement associated with rotation of the fragment. In this reported case, the lateral condyle fracture was Milch type II and Stage III.

The fixation of lateral condyle fracture is of prime importance as it constitutes Salter Harris type IV injury. The evidence supports prompt open reduction and internal stabilisation of the lateral humeral condyle fracture to provide the best results [3,8,10]. Growth plate and articular surface should be aligned and restored. Missing or inadequately treated lateral humeral condylar fracture can lead to non-union, malunion, recurrent dislocation, abnormalities in carrying angle, prominence of the lateral humeral condyle, progressive cubitus valgus deformity and tardy ulnar palsy [8,10,11].

The main focus has been changed more recently from medial to lateral collateral ligament for traumatic elbow instability in adults. Lateral collateral ligament acts as a major elbow stabilizer and is predominantly responsible for acute recurrent and chronic recurrent dislocations. In children, a lateral capsular pocket defect with an ununited lateral epicondylar fragment has been described contributing to recurrent elbow dislocation and instability. Fixation of this small fragment restores the lateral collateral ligament integrity and improves elbow stability [13]. Lateral condyle or epicondyle fracture may represent variations of lateral collateral ligament injury. We would like to confirm here that our case had an elbow dislocation variant with lateral condyle fracture instead of avulsion or intrasubstance tear of lateral collateral ligament complex.

Van Haaren et al [3] reported a rare combination of injury in the form of pediatric posterior elbow dislocation with an associated Milch Type II lateral condyle fracture, while Murnaghan et al [6] and Rovinsky et al [7] described the Milch Type I lateral condyle fracture-dislocation. These authors recommended oblique, heterolateral and varus stress films to aid in diagnosis. Evidence confirms that if a dislocated elbow is associated with a fracture, it is essential to reduce the dislocation first. After assessing the fracture on the post-reduction films, the fracture can be treated as if the dislocation had not occurred [3]. In our case, the elbow dislocation was reduced by closed manipulation and subsequently the elbow stability was assessed under general anaesthesia. Open reduction and internal fixation of lateral condyle fracture was carried out with the help of two Kirschner wires. The patient had an uneventful recovery with an excellent outcome as per Hardacre functional rating system for evaluation of the results (i.e. no loss of motions, no alteration in the carrying angle and no symptoms).

In cases of elbow fracture-dislocation, prompt reduction of the elbow dislocation is essential. Anatomic reduction and fixation of the lateral condylar fracture fragment can provide stability for the elbow and improve the functional result. This report presents a very rare condition. As these fractures are quite unusual to encounter, the management of such fractures can be technically demanding. In summary, concomitant elbow dislocation should be managed by closed reduction followed by same day or next day anatomical open reduction and cannulated screw fixation or K-wiring of the lateral condyle fixation. Postoperatively, a close clinico-radiologic follow-up helps in early diagnosis of the loss of reduction.

## Conclusion

In summary, complex pediatric elbow injuries are quite unusual to encounter, the management of such fractures can be technically demanding. Concomitant elbow dislocation can be managed by closed reduction followed by open K-wiring or cannulated screw fixation of the lateral condyle fracture. Postoperatively, a close clinico-radiological follow-up helps in early diagnosis of the loss of reduction. We recommend that all patients with a dislocated elbow should have elbow stability assessed under general anaesthesia because of high complication rate associated with a missed lateral condylar injury.

## Competing interests

The author(s) declare that they have no competing interests.

## Authors' contributions

Each author has equally contributed. HS collected the data and written up the manuscript, RA helped in scrutiny of the paper and obtaining illustrations. GRT had the idea and granted permission to use his patient data for preparing the manuscript.

## Pre-publication history

The pre-publication history for this paper can be accessed here:


